# Targeting the stem cell niche: role of collagen XVII in skin aging and wound repair

**DOI:** 10.7150/thno.78016

**Published:** 2022-09-06

**Authors:** Yangdan Liu, Chiakang Ho, Dongsheng Wen, Jiaming Sun, Lu Huang, Ya Gao, Qingfeng Li, Yifan Zhang

**Affiliations:** Department of Plastic & Reconstructive Surgery, Shanghai Ninth People's Hospital, School of Medicine, Shanghai Jiao Tong University, Shanghai, China.

**Keywords:** Collagen XVII, Aging, Wound repair, Stem cell, Stem cell niche

## Abstract

The skin epidermis and appendages undergo ongoing renewal throughout life. Stem cells residing in the epidermis and hair follicles are pivotal for sustaining skin homeostasis. The self-renewal ability of stem cells significantly decreases during skin aging but actively increases during wound repair. Residential stem cells reside in niches that provide spatially distinct microenvironments for stem cell maintenance and function. Cell-extracellular matrix (ECM) adhesion is essential for the establishment of niche architecture. Collagen XVII (COL17), as a transmembrane protein constituting hemidesmosomes (HDs), mediates the interactions of stem cells with surrounding cells and the matrix to regulate skin homeostasis, aging and wound repair. This review focuses on the pivotal role of the niche component COL17 in stem cell maintenance and its function in regulation of skin aging and wound repair.

## 1. Brief introduction of COL17

The transmembrane protein collagen XVII (COL17/BP180/BPAG2) is a structural component of hemidesmosomes (HDs). It was first named BP180 due to its initial discovery in bullous pemphigoid (BP) in the 1980s 1, 2, and the sequence of the protein was then identified in the early 1990s 3. BP180 was then relabelled as collagen XVII, as several collagenous domains consisting of repeating triplet glycine-X-Y were detected in it 4. Since then, a series of studies have revealed the structure of this protein. It has a globular intracellular domain (ICD) and a large extracellular domain (ECD) containing collagenous domains interspersed with noncollagenous domains 3, 5. The assembly of the collagen triple helix forms the higher architecture of a trimer, which undergoes constitutive or induced shedding 6.

COL17 is mainly expressed by basal keratinocytes, and its ICD binds to keratin intermediate filaments through plectin and BP230 7, while the ECD binds to integrin α6, laminin-332, and collagen IV 8-10. These multiprotein complexes form HDs at the dermal-epidermal basement membrane zone, mediating the adhesion of keratinocytes to the underlying membrane 11. Another kind of COL17, defined as non-HD COL17 present in the apicolateral portion of basal keratinocytes, may play other roles in keratinocyte physiology 12.

COL17 has been reported to be involved in multiple skin diseases. In autoimmune skin diseases, such as BP, mucous membrane pemphigoid, gestational pemphigoid, and linear IgA bullous dermatosis, COL17 acts as an autoantigen 13-16. COL17 is deficient in hereditary blistering skin diseases, such as intermediate junctional epidermolysis bullosa (JEB) 17. In addition, it also plays a role in skin cancers, such as squamous cell carcinoma, basal cell carcinoma, and malignant melanocytic tumors 18-20.

Since the skin of JEB patients is also characterized by skin atrophy and fragility, alopecia, dyspigmentation, and delayed wound healing 21, 22. The functions of COL17 in skin aging and wound repair have been gradually revealed in nondiseased skin. Intriguingly, COL17 often works by regulating stem cells. Here, we provide an overview of COL17 in skin aging and skin wound repair with special emphasis on its role in stem cell niches. We begin with the introduction to COL17 and the role of stem cells in skin aging and wound repair. We then discuss the effect of COL17 on the stem cell niches of epidermal stem cells (ESCs), hair follicle stem cells (HFSCs), and melanocyte stem cells (MSCs) in skin aging and the involvement of COL17 in ESC population dynamics and motility in skin wound repair. In addition, we discuss the clinical implications of COL17 for antiaging and wound repair. Ultimately, we propose future perspectives for research on COL17.

## 2. COL17 as a critical modulator in skin aging

### 2.1 Stem cells in skin aging

**Figure 1:**
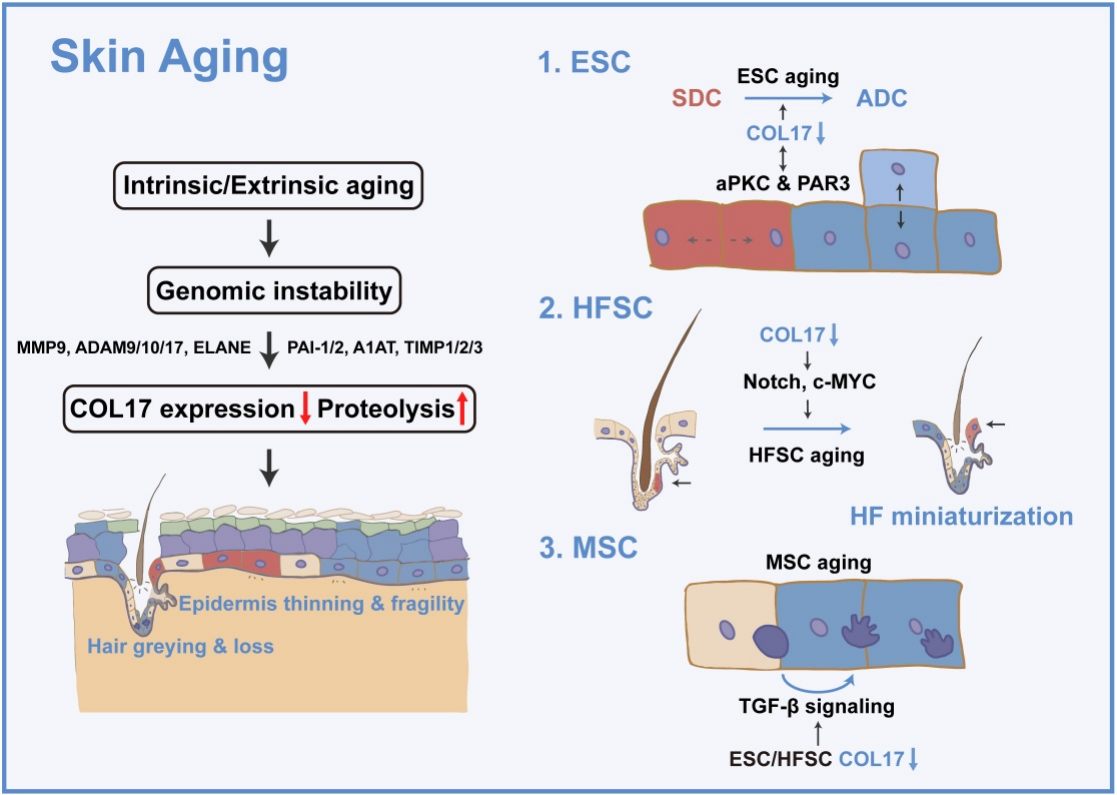
** COL17 as a critical modulator in skin aging.** Intrinsic or extrinsic aging leads to genomic instability and then results in decreased COL17 expression and increased COL17 proteolysis, which is regulated by proteases (e.g., MMP9, ADAM9, 10, 17, ELANE) and protease inhibitors (e.g., PAI-1, PAI-2, A1AT, TIMP1, TIMP2, TIMP3). These finally induce epidermis thinning and fragility, and hair greying and loss. For ESCs, COL17 down-regulation leads to the imbalance of SDCs and ADCs through interactions with aPKC and PAR3. For HFSCs, down-regulation of COL17 causes HF miniaturization through Notch and c-MYC signaling. For MSCs, down-regulation of COL17 in ESCs and HFSCs creates a niche with the involvement of TGF-β signaling, leading to MSC aging.

The skin shows profound structural and functional changes with age, including epidermal and dermal thinning, loss of dermal elasticity and wrinkling, and greying and loss of hair 23. Skin aging is induced by intrinsic aging, also known as chronological aging, or by extrinsic aging through environmental factors, such as air pollution and ultraviolet (UV) light 24. Damage theory is a widely accepted mechanism of acceleration of skin aging and involves accumulation of DNA damage by replication errors, reactive oxygen species, eroded telomeres, and chromosome breaks 25. Tissue decline due to genomic instability has been explained by cellular senescence or apoptosis 25. Nevertheless, the dynamics of the constituent cells and their cellular fate and determination of whether aged or damaged cells accumulate or are eliminated in tissues and organs during the aging process have always been an intractable problem 25. Adult stem cells are vital for replacing cells in tissues, yet their capacity declines with age 26. Hence, stem cells in the skin, including ESCs, HFSCs, and MSCs, are prospective keys to cell dynamics in skin aging. These adult stem cells reside in niches that provide spatially distinct microenvironments for stem cell maintenance and function. The conceptual framework for stem cell niches, their compositions, and their operating logistics is constantly being updated throughout life; thus, the stem cell niche has been considered to contribute to the 'fountain of youth' 23, 27-29. In the following section, the regulatory role of COL17 in multiple skin stem cells will be discussed (Figure 1).

### 2.2 COL17 in ESCs

Accumulating evidence has confirmed that cell-cell and cell-extracellular matrix adhesion is essential for the establishment and maintenance of niche architecture 30. Adhesion to the underlying extracellular matrix has been suggested as an important factor in ESC maintenance 31, 32. Hemidesmosomes are diminished by aging and result in the microdelamination of basal cells 33, 34. Among all HD components, COL17 is the only one that shows a significant decrease during aging 35, 36. Subsequent studies have demonstrated that hemidesmosome instability is caused by the proteolysis of COL17A1 induced by genomic instability both by intrinsic aging 37 and UVB-induced photoaging 38. Accumulating studies have already elucidated the roles of proteases and protease inhibitors in COL17A1 proteolysis, including proteases MMP9, ADAM9, 10, 17, and ELANE 39-42, as well as protease inhibitors PAI-1, PAI-2, A1AT, TIMP1, TIMP2, and TIMP3 43-47.

The exact function of COL17 in aging was recently reported 37. In skin homeostasis, COL17A1 is differentially expressed in different basal cell clones, and COL17A1^high^ ESCs are constantly outcompeting COL17A1^low^ cells to eliminate stressed or unfit cells from the skin. Lineage tracing for aged skin presents an expanding single-type cell clone consisting of noncompetitive COL17A1^-^MCM2^-^ cells in the basal layer instead of the conspicuous heterogeneity of COL17A1 expression in young skin. Cell competition is possibly driven by the two types of cell divisions mediated by differential COL17A1 expression. COL17A1^low^ cells exhibit an increased ratio of perpendicular cell divisions to generate a basal cell and an apically located differentiating suprabasal cell, whereas COL17A1^high^ cells divide in parallel to generate two identical basal daughter cells. Therefore, the COL17-mediated symmetric cell divisions (SCDs) mechanistically push out COL17A1^low^ cells by causing a reduced number of hemidesmosomes allowing for microdetachment from the basement membrane. In addition to the cell competition theory, COL17 can directly increase the self-renewal capacity of epidermal cell colonies by increasing the ratio of parallel divisions 48. Moreover, the reduction in non-HD COL17 results in increased asymmetric cell divisions (ACDs) of ESCs, leading to abnormally increased epidermal stratification in the paws of aged mice 49. These results indicate that both HD COL17 and non-HD COL17 are crucial for the regulation of cell divisions in ESCs, and the balance of SCDs and ACDs is particularly critical for regulating skin homeostasis and is disturbed during skin aging.

The mechanism by which COL17 regulates cell division is discussed in subsequent studies 49. Aging-induced changes in the intracellular calcium concentration lead to the inhibition of atypical PKC (aPKC), a confirmed stem cell division orientation regulator, thus diminishing apicolateral COL17 in basal cells. COL17, in turn, interacts with a complex formed by aPKC and proteinase-activated receptor 3 (PAR3), a cell polarity regulator for stem cell maintenance 12, 50. Collectively, the reciprocal regulation between aPKC and COL17 alters cell polarity, promoting ASD in epidermal cells and increasing terminally differentiated cells in aged skin. Intriguingly, the effect of PKC signaling on COL17 is controversial. In contrast to the above modification mechanism, non-HD COL17 phosphorylation and endocytosis are induced by PKC 51, and HD COL17 destabilization is driven by aPKC 52, which is attributed to the different upstream signaling pathways of PKC activation.

Collectively, age-induced COL17 proteolysis leads to the imbalance of SCDs and ACDs in ESCs through PKC signaling, resulting in the loss of cell competition, self-renewal capacity, and stem cell maintenance, ultimately causing age-associated epidermal atrophy and fragility and nonhealing wounds, which will be discussed in the following section.

### 2.3 COL17 in HFSCs

Hair loss is one of the common manifestations of skin aging 53. The hair follicle (HF) is an epithelial mini-organ of the skin that sustains cyclic hair regrowth over repeated hair cycles. HFSCs are responsible for the cyclic regeneration of hair follicles and also serve as a transient supply of progeny to the interfollicular epidermis (IFE) and sebaceous glands after wounding 54. HFSCs receive signals from their surroundings and actively send out signals to modulate the organization and function of their own niches 55.

HF aging features miniaturization of hair follicles and the absence of dermal papillae, sebaceous glands, and the infundibulum. Preceding HF miniaturization is stem cell dysregulation, including the loss of HFSC markers and HFSC maintenance markers. Similar to ESCs, HFSCs are committed to epidermal terminal differentiation and keratinization during aging; these cells are distributed in the junctional zone located above the bulge area; they then move to the suprabasal epidermis and eventually to the skin surface to be eliminated from the stem cell pool and from the skin 25.

The expression of COL17 in HFSCs has been observed. COL17 is specifically reduced in aged quiescent HFSCs, and the expression and distribution of non-HD COL17A1 in HFSCs changes greatly during aging, negating the contribution of HD COL17-induced skin detachment in hair loss 27. From the perspective of mechanism, COL17 suppresses the “HFSC aging state” by inhibiting the loss of stem cell signature and epidermal commitment, thereby enabling HFSCs to maintain HFSC quiescence and immaturity. The epidermal differentiation regulators Notch and c-MYC are potential targets of non-HD COL17, but this requires further verification 25. Moreover, the maintenance of COL17A1 is not only indispensable for HFSC maintenance but is also effective for the protection of HFSCs against “HFSC aging” and resultant “HF aging” characterized by HF miniaturization, hair loss, and skin thinning.

### 2.4 COL17 in MSCs

MSCs are pigment-producing melanocytes residing around ESCs and HFSCs in the interfollicular epidermis or the follicular bulge-subbulge area 56. They are closely related to the hypopigmentation of skin and hair follicles in the process of intrinsic and extrinsic aging 27, 37.

COL17 deficiency induces relatively mild dyspigmentation in the tail skin, as well as hair greying. However, COL17 is not expressed in MSCs, indicating the indirect effect of COL17 on MSCs. As envisaged, differentiated melanocytes colocated with differentiated ESCs and HFSCs exist early in preaging skin and hair follicles, owing to the niche created by ESCs and HFSCs through TGF-β signaling 27. This further confirms the role of COL17 in creating niches for surrounding stem cells.

## 3. COL17 as a crucial regulator in wound repair

### 3.1 Stem cells in wound repair

Wound healing occurs through distinct overlapping phases: hemostasis, inflammation, proliferation, and remodeling 57. Re-epithelialization is a vital physiological process in the proliferative phase; it describes the resurfacing of a wound with new epithelium and is critical for restoring barrier function 58. During skin homeostasis, the skin epithelium renews throughout life in a continuous turnover ensured by stem cells that balance proliferation and differentiation to replace dead and terminally differentiated cells 59, 60. During wound repair, stem cells are activated and recruited from different skin regions, and the vacant niche created by injury activates a broad range of stem cells to assume characteristics that differ from their homeostatic roles.

Stem cells are mainly involved in three biological processes during re-epithelialization, including stem cell migration and proliferation, stem cell population dynamics, and stem cell plasticity 59. First, epidermal injury is typically followed by increased keratinocyte migration and proliferation 57. Interestingly, keratinocytes do not proliferate but migrate as a cellular sheet at the leading edge, which is surrounded by a proliferation zone at a distance away from the edge 61, 62. In addition, increasing symmetric renewal or decreasing differentiation of cells compensates for lost cells during re-epithelialization, whereas lineage hierarchy and the balance between self-renewal and differentiation of committed progenitors remain unchanged from their states during homeostasis 59. Ultimately, plasticity upon wound healing is observed in different skin lineages. HFSCs progressively lose their initial identity and are reprogrammed to an IFE fate when recruited to the IFE upon injury 63, and differentiated suprabasal epidermal cells are able to revert to a stem cell state upon wounding 64, 65.

**Figure 2:**
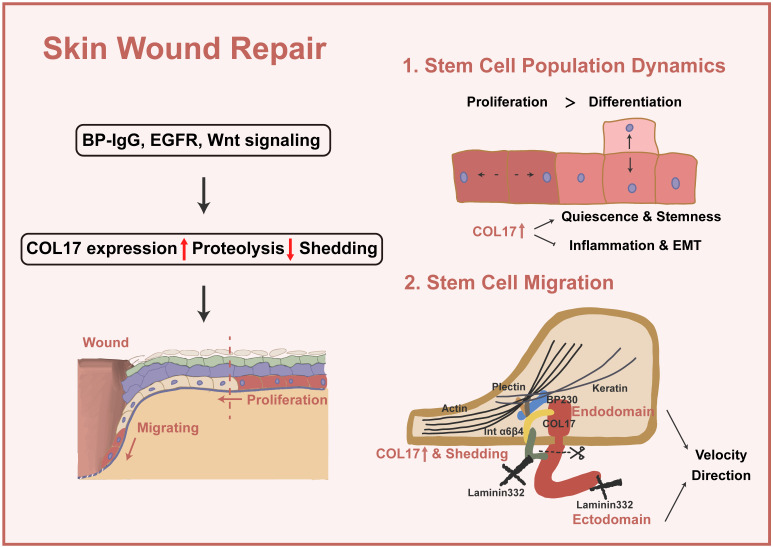
** COL17 as a crucial regulator in wound repair.** Several signalings (e.g., BP-IgG, EGFR and Wnt signaling) lead to increased COL17 expression, decreased COL17 proteolysis, and COL17 shedding. Then, up-regulation of COL17 regulates stem cell population dynamics and migration. For stem cell population dynamics, up-regulation of COL17 represents a higher level of cell quiescence and stemness, as well as inflammation and EMT. For stem cell migration, up-regulation and shedding of COL17 regulate migration velocity and directions through interactions with other intracellular or ECM components.

Apart from preaging of skin, decreased wound closure is induced by COL17 KO and increased wound closure by inhibition of COL17 shedding 37, 66, 67. In the following section, the pivotal role of COL17 in wound repair through modulation of stem cell migration and proliferation and stem cell population dynamics will be discussed (Figure 2).

### 3.2 COL17 in stem cell population dynamics

In single-cell analysis research 68, a COL17A1^high^ subcluster with top markers, such as COL17A1 and TP63, a gene enriched in quiescent bulge HF stem cells, is present in both wounded and unwounded skin. The COL17A1^high^ state scores the lowest for inflammation and EMT genes but the highest for genes of a “quiescence and sternness” signature derived from tissue quiescent stem cells, which are more quiescent, persist longer and could give rise to more rapidly cycling committed progenitors with a shorter lifespan. This portion of cells differentiates directly or indirectly into spinous cells or a specific pool of proliferating basal cells, thus maintaining skin renewal during homeostasis. In wound repair, such cells differentiate into suprabasal cells at the wound periphery or convert into cells of higher motility and subsequently migrate into the wound.

Two kinds of colonies are formed in human keratinocyte culture, expanding colonies and stacking colonies 48; the former mainly displays proliferating/proliferating divisions, while the latter presents more differentiating/differentiating divisions. COL17A1 is highly expressed in cells of expanding colonies, and the inhibition of COL17A1 decreases the clonal growth of keratinocytes and increases the ratio of the stacking colony type in the culture. Together, these discoveries imply that cells with high expression of COL17 show excessive renewal over differentiation, which helps compensate for lost cells during re-epithelialization.

### 3.3 COL17 in stem cell migration

The migration of epidermal basal cells requires the remodeling of the cell-cell and cell-substratum contacts to allow the cells to detach from the intact basement membrane in the unwounded epidermis. As the cells move over the wound, they degrade the provisional matrix while depositing new matrix proteins, including laminin-332. In keratinocytes, matrix proteins and their receptors are clustered into two distinct protein complexes: focal adhesions and hemidesmosomes 69. Focal adhesions are understood to be dynamic attachment points with roles in cell spreading and motility 70, yet HDs are traditionally viewed as attachment complexes that promote stable adhesion of basal epithelial cells in the resting epidermis. HDs are currently recognized to be disassembled to allow keratinocytes to move onto the wound bed and/or over the provisional wound matrix and play important regulatory and signaling roles in determining aspects of the motile behavior of skin cells. In the detection of the wound edge, the Day 3 wound edge shows a strongly elevated level of COL17 throughout the whole wound epithelium, including the distal regions behind the wound margin as well as the growing epithelial tongues; in contrast, at Day 6, COL17 is mainly localized along the growing epithelial tongues 67, 71. When exploring the role of COL17 in migration, it is confusing to find that it regulates different characteristics of cell motility through diverse mechanisms. Therefore, we classified the different functions of COL17 in these studies to clarify whether these findings are controversial or represent the multifunctional role of COL17.

The features of cell motility can be roughly grouped into the velocity and the directions of migration 72, and COL17 has both a positive effect 73 and a negative effect 8 on cell velocity. Changes in actin dynamics are often observed in these studies. It is worth noting that the knockout of COL17 by different methods has different effects. Primary keratinocytes from COL17 KO transgenic mice show increased migration speed, whereas cell lines knocked down with shCOL17 display a decrease in migration speed, owing to the different compensatory responses of other HD proteins in response to this different method of COL17 KO. The protein level of β4 integrins is significantly induced or the structural impediment of β4 integrin movement is removed in response to COL17 KO in skin and keratinocytes, but further investigations are needed to clarify this dynamic regulation 74. In contrast, knockdown with shCOL17 only changes cell motility exclusive to cell adhesion, indicating a lack of compensatory response 73.

For studies in which the velocity shows no correlation with COL17, the directions of migration are considered. To migrate efficiently in a directed manner, cells must establish and maintain an asymmetric morphology with defined leading and trailing edges 72. The dysregulation of migration direction resulting from COL17 KO significantly slows down the process of wound closure 74-76, and this is accompanied by formation of destabilized lamellipodium, the key organelle that generates the force necessary for directional protrusion at the cell periphery.

In addition to the different objects of regulation, whether COL17 itself or COL17 shedding plays a role in wound repair has been discussed. A scenario can be visualized in the wound in which shedding of the COL17 ectodomain loosens or releases the cell from some of its present binding partners and allows it to embark on other functions, as required 9, 39. The ectodomain-selective staining increases along with the leading epithelial tongue, and the nonshedding COL17 skin shows accelerated motility, especially enhanced velocity 67; however, the directionality of migration is nearly twofold lower than that in normal skin, which is accompanied by the lack of stable polarization with lamellipodium. Here, COL17 ectodomain shedding acts as a cell-intrinsic repressor of mTOR-controlled keratinocyte migration speed and proliferation 66.

Thus far, it is not clear whether the lack of soluble ectodomain, the concomitantly generated membrane-tethered endodomain of COL17 or the combination of both caused accelerated re-epithelialization in nonshedding COL17 skin. There is evidence that the released ectodomain has a direct suppressive effect on keratinocyte motility because 1) deposits of the highly stable released COL17 ectodomain are mainly found in the trails of migrating cells incorporated with laminin-332, leading to stabilization of the substrate and cell immobilization but directed cell motility 8, 9, 75, 77, 78 and 2) the addition of purified soluble ectodomain reduced keratinocyte migration in scratch-wound assays 39. In another study, the endodomain signal was completely lost in the protrusions at the leading and trailing cell edges of nonshedding COL17 keratinocytes, whereas no changes were evident in the ectodomain, indicating that the absence of the endodomain stump in migrating keratinocytes leads to unstable rear-to-front polarization and altered laminin-332 deposition 66 and that the addition of the COL17 endodomain rescues lamellipodium dynamics and motile behavior 74. The COL17 endodomain regulates migration through complex interactions with the cytoskeleton. Altered organization of actin is triggered by a deficiency of COL17, and COL17 mediates the association of BPAG1e and α6β4 integrin, modulating RAC1 activation, an important regulator of the actin filaments in lamellipodium 74. COL17 also stabilizes the actin4 interaction at the substrate-attached surface by binding to α6β4 integrin 79. A recent study reported that COL17 mediates both actin and keratin dynamics through plectin 48. However, the disordered cytoskeleton in turn induces the distribution of α6β4 integrin 66.

In terms of the upstream regulatory mechanism of COL17, BP-IgG in BP induces the internalization of COL17 to inhibit its function and reduce cell motility 80, and the aging-induced decline of EGFR inhibits the proteolysis of COL17 to regulate cell motility 48. It has been confirmed that Wnt signaling affects COL17, which is related to wound repair 52.

## 4. COL17 as a potential therapeutic target for skin aging and wound repair

As introduced above, the active restoration of stem cell pools by the use of residual intact stem cells before the irreversible advancement of tissue architectural changes may be essential for successful antiaging and tissue regeneration in the skin. COL17, as a good marker for stem cells reflecting individual cellular potential and quality for self-renewal, represents an interesting therapeutic target in antiaging and wound treatment.

An *in vitro* study showed that the forced maintenance of COL17 rescues both epidermal thinning and hair loss in aged mice for more than 24 months, suggesting that the stimulation of COL17 expression is a potential therapy for skin aging 25, 37. Therefore, a novel kind of peptide derivative has been synthesized to stimulate the synthesis of BM proteins, including COL17 and laminins. Its antiaging effect has been clinically verified (P1910-664), showing significant improvements in facial wrinkles 81. In another study, with the combined use of radiofrequency and ultrasound techniques, there was a huge increase in COL17 and other matrices in human skin substitutes, suggesting another potential therapy for skin aging 82.

For patients with COL17 deficiency, genetic therapy has been introduced to solve the skin aging problem 83, 84. Fortunately, a group of spontaneously genetically corrected cells with normal expression of COL17 have been found in the skin of these patients. These reverent cells were reprogrammed into iPSCs, and their differentiation into the keratinocyte lineage was induced. Revertant-iPSC keratinocytes were obtained and used to reconstitute skin with marked functional and structural recovery and rejuvenation.

Regarding wound repair, forced expression of COL17 by basal keratinocytes in mice promotes wound healing 37. Encouragingly, two chemicals, Y27632 and apocynin, have been identified to induce COL17 expression in keratinocytes, which then increases the self-renewing capabilities of ESCs. In addition, the application of these drugs to full-thickness skin wounds significantly promotes wound repair, but this requires further clinical verification in human skin wounds 37.

Collectively, upregulation of COL17 is a common solution for skin rejuvenation and wound healing. Therefore, studies on the mechanism of pre- and posttranscriptional regulation of COL17 set the basis for more therapeutic strategies. Moreover, the development of synthetic peptides that stimulate COL17 synthesis and high-throughput screening for drugs that upregulate COL17 and subsequent clinical trials of such drugs will promote the clinical translation of these findings.

## 5. Future perspective

Taken together, this body of work provides previously unappreciated insights into the cellular and molecular mechanisms by which COL17 regulates skin aging and wound repair, focusing on the perspective of stem cell niches.

However, many open questions remain regarding the detailed upstream mechanisms modulating COL17 level. We know little about the signaling pathway regulating the gradual or rapid decline of COL17 during aging and the high expression and distribution of COL17 to the wound edges. Downstream mechanisms also remain to be discovered. It is also necessary to elucidate how the multiple mechanisms introduced above interact with each other; for example, whether the functions of HD COL17 and non-HD COL17 overlap or are independent and the paradoxical role of COL17 in the regulation of cell migration has not yet been verified. In addition to the deepening of existing studies, the research direction can be further broadened. For instance, considering the regulation of COL17 on directed cell migration and its distribution to the wound edge, it will be interesting to further detect the potential role of COL17 in collective cell migration. Moreover, due to the dynamic change in COL17 expression and the return of cells in differentiated states to the COL17A1^high^ state in lineage tracing of wounded skin, the exploration of the function of COL17 in stem cell plasticity will be an important challenge for the future.

When the functions and mechanisms of COL17 are fully studied and corresponding clinical trials are performed, it will be possible to propose future therapeutic strategies for skin aging and wound repair.

## Abbreviations

COL17: collagen XVII
HD: Hemidesmosome
BP: bullous pemphigoid
ICD: intracellular domain
ECD: extracellular domain
HFSC: hair follicle stem cell
MSC: melanocyte stem cell
UV: ultraviolet
MMP: matrix metallopeptidase
ADAM: a disintegrin and a metalloprotease
ELANE: neutrophil elastase
PAI: plasminogen activator inhibitor
A1AT: alpha-1-antitrypsin
TIMP: tissue inhibitor of metalloproteinases
MCM2: minichromosome maintenance protein
SCD: symmetric cell division
ACD: asymmetric cell divisions
KO: knockout
aPKC: atypical protein kinase C
PAR3: proteinase-activated receptor 3
PDGFRa: platelet-derived growth factor regulator a
CCN1: cysteine-rich 61, connective tissue growth factor, and nephroblastoma 1
HF: hair follicle
IFE: interfollicular epidermis
TGF-β: transforming growth factor-β
RAC1: RAS-related C3 botulinum toxin substrate 1
EGFR: epidermal growth factor receptor

## References

[1] Stanley JR, Woodley DT, Katz SI (1984). Identification and partial characterization of pemphigoid antigen extracted from normal human skin. J Invest Dermatol.

[2] Labib RS, Anhalt GJ, Patel HP, Mutasim DF, Diaz LA (1986). Molecular heterogeneity of the bullous pemphigoid antigens as detected by immunoblotting. J Immunol.

[3] Giudice GJ, Emery DJ, Diaz LA (1992). Cloning and primary structural analysis of the bullous pemphigoid autoantigen BP180. J Invest Dermatol.

[4] Li K, Tamai K, Tan EM, Uitto J (1993). Cloning of type XVII collagen. Complementary and genomic DNA sequences of mouse 180-kilodalton bullous pemphigoid antigen (BPAG2) predict an interrupted collagenous domain, a transmembrane segment, and unusual features in the 5'-end of the gene and the 3'-untranslated region of the mRNA. J Biol Chem.

[5] Hirako Y, Usukura J, Nishizawa Y, Owaribe K (1996). Demonstration of the molecular shape of BP180, a 180-kDa bullous pemphigoid antigen and its potential for trimer formation. J Biol Chem.

[6] Nishie W, Jackow J, Hofmann SC, Franzke CW, Bruckner-Tuderman L (2012). Coiled coils ensure the physiological ectodomain shedding of collagen XVII. J Biol Chem.

[7] Natsuga K, Nishie W, Nishimura M, Shinkuma S, Watanabe M, Izumi K (2017). Loss of interaction between plectin and type XVII collagen results in epidermolysis bullosa simplex. Hum Mutat.

[8] Tasanen K, Tunggal L, Chometon G, Bruckner-Tuderman L, Aumailley M (2004). Keratinocytes from patients lacking collagen XVII display a migratory phenotype. Am J Pathol.

[9] Nishie W, Kiritsi D, Nystrom A, Hofmann SC, Bruckner-Tuderman L (2011). Dynamic interactions of epidermal collagen XVII with the extracellular matrix: laminin 332 as a major binding partner. Am J Pathol.

[10] Koster J, Geerts D, Favre B, Borradori L, Sonnenberg A (2003). Analysis of the interactions between BP180, BP230, plectin and the integrin alpha6beta4 important for hemidesmosome assembly. J Cell Sci.

[11] McMillan JR, Akiyama M, Shimizu H (2003). Epidermal basement membrane zone components: ultrastructural distribution and molecular interactions. J Dermatol Sci.

[12] Watanabe M, Kosumi H, Osada SI, Takashima S, Wang Y, Nishie W (2021). Type XVII collagen interacts with the aPKC-PAR complex and maintains epidermal cell polarity. Exp Dermatol.

[13] Bağcı IS, Horváth ON, Ruzicka T, Sárdy M (2017). Bullous pemphigoid. Autoimmun Rev.

[14] Huilaja L, Hurskainen T, Autio-Harmainen H, Hofmann SC, Sormunen R, Räsänen J (2008). Pemphigoid gestationis autoantigen, transmembrane collagen XVII, promotes the migration of cytotrophoblastic cells of placenta and is a structural component of fetal membranes. Matrix Biol.

[15] Xu HH, Werth VP, Parisi E, Sollecito TP (2013). Mucous membrane pemphigoid. Dent Clin North Am.

[16] Cozzani E, Di Zenzo G, Gasparini G, Salemme A, Agnoletti AF, Vassallo C (2020). Autoantibody Profile of a Cohort of 54 Italian Patients with Linear IgA Bullous Dermatosis: LAD-1 Denoted as a Major Auto-antigen of the Lamina Lucida Subtype. Acta Derm Venereol.

[17] Has C, Bauer JW, Bodemer C, Bolling MC, Bruckner-Tuderman L, Diem A (2020). Consensus reclassification of inherited epidermolysis bullosa and other disorders with skin fragility. Br J Dermatol.

[18] Yamada T, Endo R, Tsukagoshi K, Fujita S, Honda K, Kinoshita M (1996). Aberrant expression of a hemidesmosomal protein, bullous pemphigoid antigen 2, in human squamous cell carcinoma. Lab Invest.

[19] Krenacs T, Kiszner G, Stelkovics E, Balla P, Teleki I, Nemeth I (2012). Collagen XVII is expressed in malignant but not in benign melanocytic tumors and it can mediate antibody induced melanoma apoptosis. Histochem Cell Biol.

[20] Parikka M, Kainulainen T, Tasanen K, Bruckner-Tuderman L, Salo T (2001). Altered expression of collagen XVII in ameloblastomas and basal cell carcinomas. J Oral Pathol Med.

[21] McGrath JA, Gatalica B, Christiano AM, Li K, Owaribe K, McMillan JR (1995). Mutations in the 180-kD bullous pemphigoid antigen (BPAG2), a hemidesmosomal transmembrane collagen (COL17A1), in generalized atrophic benign epidermolysis bullosa. Nat Genet.

[22] Jonkman MF, de Jong MC, Heeres K, Pas HH, van der Meer JB, Owaribe K (1995). 180-kD bullous pemphigoid antigen (BP180) is deficient in generalized atrophic benign epidermolysis bullosa. J Clin Invest.

[23] Hsu YC, Li L, Fuchs E (2014). Emerging interactions between skin stem cells and their niches. Nat Med.

[24] Franco AC, Aveleira C, Cavadas C (2022). Skin senescence: mechanisms and impact on whole-body aging. Trends Mol Med.

[25] Matsumura H, Mohri Y, Binh NT, Morinaga H, Fukuda M, Ito M (2016). Hair follicle aging is driven by transepidermal elimination of stem cells via COL17A1 proteolysis. Science.

[26] Shirai K, Obara K, Tohgi N, Yamazaki A, Aki R, Hamada Y (2019). Expression of anti-aging type-XVII collagen (COL17A1/BP180) in hair follicle-associated pluripotent (HAP) stem cells during differentiation. Tissue Cell.

[27] Tanimura S, Tadokoro Y, Inomata K, Binh NT, Nishie W, Yamazaki S (2011). Hair follicle stem cells provide a functional niche for melanocyte stem cells. Cell Stem Cell.

[28] Li L, Xie T (2005). Stem cell niche: structure and function. Annu Rev Cell Dev Biol.

[29] Morrison SJ, Spradling AC (2008). Stem cells and niches: mechanisms that promote stem cell maintenance throughout life. Cell.

[30] Raymond K, Deugnier MA, Faraldo MM, Glukhova MA (2009). Adhesion within the stem cell niches. Curr Opin Cell Biol.

[31] Watt FM (2002). Role of integrins in regulating epidermal adhesion, growth and differentiation. Embo j.

[32] Green H (1977). Terminal differentiation of cultured human epidermal cells. Cell.

[33] Le Varlet B, Chaudagne C, Saunois A, Barré P, Sauvage C, Berthouloux B (1998). Age-related functional and structural changes in human dermo-epidermal junction components. J Investig Dermatol Symp Proc.

[34] Dos Santos M, Metral E, Boher A, Rousselle P, Thepot A, Damour O (2015). *In vitro* 3-D model based on extending time of culture for studying chronological epidermis aging. Matrix Biol.

[35] Park S, Kang S, Lee WJ (2021). Menopause, Ultraviolet Exposure, and Low Water Intake Potentially Interact with the Genetic Variants Related to Collagen Metabolism Involved in Skin Wrinkle Risk in Middle-Aged Women. Int J Environ Res Public Health.

[36] Langton AK, Halai P, Griffiths CE, Sherratt MJ, Watson RE (2016). The impact of intrinsic ageing on the protein composition of the dermal-epidermal junction. Mech Ageing Dev.

[37] Liu N, Matsumura H, Kato T, Ichinose S, Takada A, Namiki T (2019). Stem cell competition orchestrates skin homeostasis and ageing. Nature.

[38] Xiang Y, Liu Y, Yang Y, Yan Y, Kim AJ, Guo C (2022). Reduced expression of Collagen 17A1 in naturally aged, photoaged, and UV-irradiated human skin *in vivo*: Potential links to epidermal aging. J Cell Commun Signal.

[39] Franzke CW, Tasanen K, Schacke H, Zhou Z, Tryggvason K, Mauch C (2002). Transmembrane collagen XVII, an epithelial adhesion protein, is shed from the cell surface by ADAMs. EMBO J.

[40] Franzke CW, Bruckner-Tuderman L, Blobel CP (2009). Shedding of collagen XVII/BP180 in skin depends on both ADAM10 and ADAM9. J Biol Chem.

[41] Hofmann SC, Voith U, Schönau V, Sorokin L, Bruckner-Tuderman L, Franzke CW (2009). Plasmin plays a role in the *in vitro* generation of the linear IgA dermatosis antigen LADB97. J Invest Dermatol.

[42] Laval S, Laklai H, Fanjul M, Pucelle M, Laurell H, Billon-Galés A (2014). Dual roles of hemidesmosomal proteins in the pancreatic epithelium: the phosphoinositide 3-kinase decides. Oncogene.

[43] Lin L, Betsuyaku T, Heimbach L, Li N, Rubenstein D, Shapiro SD (2012). Neutrophil elastase cleaves the murine hemidesmosomal protein BP180/type XVII collagen and generates degradation products that modulate experimental bullous pemphigoid. Matrix Biol.

[44] Liu Z, Zhou X, Shapiro SD, Shipley JM, Twining SS, Diaz LA (2000). The serpin alpha1-proteinase inhibitor is a critical substrate for gelatinase B/MMP-9 *in vivo*. Cell.

[45] Nishimura M, Nishie W, Shirafuji Y, Shinkuma S, Natsuga K, Nakamura H (2016). Extracellular cleavage of collagen XVII is essential for correct cutaneous basement membrane formation. Hum Mol Genet.

[46] Ståhle-Bäckdahl M, Inoue M, Guidice GJ, Parks WC (1994). 92-kD gelatinase is produced by eosinophils at the site of blister formation in bullous pemphigoid and cleaves the extracellular domain of recombinant 180-kD bullous pemphigoid autoantigen. J Clin Invest.

[47] Verraes S, Hornebeck W, Polette M, Borradori L, Bernard P (2001). Respective contribution of neutrophil elastase and matrix metalloproteinase 9 in the degradation of BP180 (type XVII collagen) in human bullous pemphigoid. J Invest Dermatol.

[48] Nanba D, Toki F, Asakawa K, Matsumura H, Shiraishi K, Sayama K (2021). EGFR-mediated epidermal stem cell motility drives skin regeneration through COL17A1 proteolysis. J Cell Biol.

[49] Watanabe M, Natsuga K, Nishie W, Kobayashi Y, Donati G, Suzuki S (2017). Type XVII collagen coordinates proliferation in the interfollicular epidermis. Elife.

[50] Ali NJA, Dias Gomes M, Bauer R, Brodesser S, Niemann C, Iden S (2016). Essential Role of Polarity Protein Par3 for Epidermal Homeostasis through Regulation of Barrier Function, Keratinocyte Differentiation, and Stem Cell Maintenance. J Invest Dermatol.

[51] Iwata H, Kamaguchi M, Ujiie H, Nishimura M, Izumi K, Natsuga K (2016). Macropinocytosis of type XVII collagen induced by bullous pemphigoid IgG is regulated via protein kinase C. Lab Invest.

[52] Kosumi H, Watanabe M, Shinkuma S, Nohara T, Fujimura Y, Tsukiyama T (2021). Wnt/beta-Catenin Signaling Stabilizes Hemidesmosomes in Keratinocytes. J Invest Dermatol.

[53] Smith L (1989). Histopathologic characteristics and ultrastructure of aging skin. Cutis.

[54] Cotsarelis G (2006). Epithelial stem cells: a folliculocentric view. J Invest Dermatol.

[55] Li KN, Tumbar T (2021). Hair follicle stem cells as a skin-organizing signaling center during adult homeostasis. Embo j.

[56] Nishimura EK, Jordan SA, Oshima H, Yoshida H, Osawa M, Moriyama M (2002). Dominant role of the niche in melanocyte stem-cell fate determination. Nature.

[57] Sun BK, Siprashvili Z, Khavari PA (2014). Advances in skin grafting and treatment of cutaneous wounds. Science.

[58] Rousselle P, Braye F, Dayan G (2019). Re-epithelialization of adult skin wounds: Cellular mechanisms and therapeutic strategies. Adv Drug Deliv Rev.

[59] Dekoninck S, Blanpain C (2019). Stem cell dynamics, migration and plasticity during wound healing. Nat Cell Biol.

[60] Blanpain C, Fuchs E (2006). Epidermal stem cells of the skin. Annu Rev Cell Dev Biol.

[61] Aragona M, Dekoninck S, Rulands S, Lenglez S, Mascré G, Simons BD (2017). Defining stem cell dynamics and migration during wound healing in mouse skin epidermis. Nat Commun.

[62] Park S, Gonzalez DG, Guirao B, Boucher JD, Cockburn K, Marsh ED (2017). Tissue-scale coordination of cellular behaviour promotes epidermal wound repair in live mice. Nat Cell Biol.

[63] Ito M, Liu Y, Yang Z, Nguyen J, Liang F, Morris RJ (2005). Stem cells in the hair follicle bulge contribute to wound repair but not to homeostasis of the epidermis. Nat Med.

[64] Fu X, Sun X, Li X, Sheng Z (2001). Dedifferentiation of epidermal cells to stem cells *in vivo*. Lancet.

[65] Mannik J, Alzayady K, Ghazizadeh S (2010). Regeneration of multilineage skin epithelia by differentiated keratinocytes. J Invest Dermatol.

[66] Jackow J, Loffek S, Nystrom A, Bruckner-Tuderman L, Franzke CW (2016). Collagen XVII Shedding Suppresses Re-Epithelialization by Directing Keratinocyte Migration and Dampening mTOR Signaling. J Invest Dermatol.

[67] Jackow J, Schlosser A, Sormunen R, Nystrom A, Sitaru C, Tasanen K (2016). Generation of a Functional Non-Shedding Collagen XVII Mouse Model: Relevance of Collagen XVII Shedding in Wound Healing. J Invest Dermatol.

[68] Haensel D, Jin S, Sun P, Cinco R, Dragan M, Nguyen Q (2020). Defining Epidermal Basal Cell States during Skin Homeostasis and Wound Healing Using Single-Cell Transcriptomics. Cell Rep.

[69] Hopkinson SB, Hamill KJ, Wu Y, Eisenberg JL, Hiroyasu S, Jones JC (2014). Focal Contact and Hemidesmosomal Proteins in Keratinocyte Migration and Wound Repair. Adv Wound Care (New Rochelle).

[70] Wozniak MA, Modzelewska K, Kwong L, Keely PJ (2004). Focal adhesion regulation of cell behavior. Biochim Biophys Acta.

[71] Gipson IK, Spurr-Michaud S, Tisdale A, Elwell J, Stepp MA (1993). Redistribution of the hemidesmosome components alpha 6 beta 4 integrin and bullous pemphigoid antigens during epithelial wound healing. Exp Cell Res.

[72] Petrie RJ, Doyle AD, Yamada KM (2009). Random versus directionally persistent cell migration. Nat Rev Mol Cell Biol.

[73] Qiao H, Shibaki A, Long HA, Wang G, Li Q, Nishie W (2009). Collagen XVII participates in keratinocyte adhesion to collagen IV, and in p38MAPK-dependent migration and cell signaling. J Invest Dermatol.

[74] Hamill KJ, Hopkinson SB, Jonkman MF, Jones JC (2011). Type XVII collagen regulates lamellipod stability, cell motility, and signaling to Rac1 by targeting bullous pemphigoid antigen 1e to alpha6beta4 integrin. J Biol Chem.

[75] Franzke CW, Has C, Schulte C, Huilaja L, Tasanen K, Aumailley M (2006). C-terminal truncation impairs glycosylation of transmembrane collagen XVII and leads to intracellular accumulation. J Biol Chem.

[76] Loffek S, Hurskainen T, Jackow J, Sigloch FC, Schilling O, Tasanen K (2014). Transmembrane collagen XVII modulates integrin dependent keratinocyte migration via PI3K/Rac1 signaling. PLoS One.

[77] Kroeger J, Hoppe E, Galiger C, Has C, Franzke CW (2019). Amino acid substitution in the C-terminal domain of collagen XVII reduces laminin-332 interaction causing mild skin fragility with atrophic scarring. Matrix Biol.

[78] Nykvist P, Tasanen K, Viitasalo T, Kapyla J, Jokinen J, Bruckner-Tuderman L (2001). The cell adhesion domain of type XVII collagen promotes integrin-mediated cell spreading by a novel mechanism. J Biol Chem.

[79] Hiroyasu S, Colburn ZT, Jones JC (2016). A hemidesmosomal protein regulates actin dynamics and traction forces in motile keratinocytes. FASEB J.

[80] Tie D, Da X, Natsuga K, Yamada N, Yamamoto O, Morita E (2019). Bullous Pemphigoid IgG Induces Cell Dysfunction and Enhances the Motility of Epidermal Keratinocytes via Rac1/Proteasome Activation. Front Immunol.

[81] Jeong S, Yoon S, Kim S, Jung J, Kor M, Shin K (2019). Anti-Wrinkle Benefits of Peptides Complex Stimulating Skin Basement Membrane Proteins Expression. Int J Mol Sci.

[82] Louis F, Fujii N, Katsuyama M, Okumoto S, Matsusaki M (2020). Effects of radiofrequency and ultrasound on the turnover rate of skin aging components (skin extracellular matrix and epidermis) via HSP47-induced stimulation. Biochem Biophys Res Commun.

[83] Umegaki-Arao N, Pasmooij AM, Itoh M, Cerise JE, Guo Z, Levy B (2014). Induced pluripotent stem cells from human revertant keratinocytes for the treatment of epidermolysis bullosa. Sci Transl Med.

[84] Pasmooij AM, Nijenhuis M, Brander R, Jonkman MF (2012). Natural gene therapy may occur in all patients with generalized non-Herlitz junctional epidermolysis bullosa with COL17A1 mutations. J Invest Dermatol.

